# Aerobic Activity in the Healthy Elderly Is Associated with Larger Plasticity in Memory Related Brain Structures and Lower Systemic Inflammation

**DOI:** 10.3389/fnagi.2016.00319

**Published:** 2016-12-26

**Authors:** Jan-Willem Thielen, Christian Kärgel, Bernhard W. Müller, Ina Rasche, Just Genius, Boudewijn Bus, Stefan Maderwald, David G. Norris, Jens Wiltfang, Indira Tendolkar

**Affiliations:** ^1^Donders Institute for Brain, Cognition and Behaviour, Radboud University NijmegenNijmegen, Netherlands; ^2^Erwin L. Hahn Institute for Magnetic Resonance Imaging, University of Essen-DuisburgEssen, Germany; ^3^Division of Forensic Psychiatry, Department of Psychiatry, Psychotherapy and Preventive Medicine, LWL-University Hospital BochumBochum, Germany; ^4^Department for Psychiatry and Psychotherapy, LVR-Hospital Essen, Faculty of Medicine, University of Duisburg-EssenEssen, Germany; ^5^Department of Psychology, University of WuppertalWuppertal, Germany; ^6^AbbVie Neuroscience DevelopmentLudwigshafen, Germany; ^7^Department of Psychiatry, Radboud University Nijmegen Medical CenterNijmegen, Netherlands; ^8^Department of Psychiatry and Psychotherapy, University Medical Center GöttingenGöttingen, Germany

**Keywords:** physical activity, elderly, memory, fMRI, functional connectivity, interleukin-6, inflammation

## Abstract

Cognitive abilities decline over the time course of our life, a process, which may be mediated by brain atrophy and enhanced inflammatory processes. Lifestyle factors, such as regular physical activities have been shown to counteract those noxious processes and are assumed to delay or possibly even prevent pathological states, such as dementing disorders. Whereas the impact of lifestyle and immunological factors and their interactions on cognitive aging have been frequently studied, their effects on neural parameters as brain activation and functional connectivity are less well studied. Therefore, we investigated 32 healthy elderly individuals (60.4 ± 5.0 *SD*; range 52–71 years) with low or high level of self-reported aerobic physical activity at the time of testing. A higher compared to a lower level in aerobic physical activity was associated with an increased encoding related functional connectivity in an episodic memory network comprising mPFC, thalamus, hippocampus precuneus, and insula. Moreover, encoding related functional connectivity of this network was associated with decreased systemic inflammation, as measured by systemic levels of interleukin 6.

## Introduction

It is a well-known phenomenon that our neurocognitive abilities change with age but there are remarkable differences in the timing and trajectory of these changes (Hedden and Gabrieli, 2004; Hofer and Alwin, 2008). Investigating the effects of lifestyle factors may be highly informative for the development of interventions to reduce or delay age-related cognitive decline. Among these lifestyle factors physical exercise both enhances and preserves cognitive function in the elderly (Dustman et al., 1984; Colcombe and Kramer, 2003; Smith et al., 2010; Bherer et al., 2013). Additionally, physical exercise appears to significantly reduce the risk of adults developing dementing diseases in later years (Laurin et al., 2001; Hamer and Chida, 2009; Middleton et al., 2010; Llamas-Velasco et al., 2015). Even patients already suffering from mild cognitive impairment or dementing disorders improve in cognitive functioning after a physical exercise intervention (Heyn et al., 2004; Lautenschlager et al., 2008). Hence, physical exercise is a promising low-cost treatment to improve neurocognitive function that is accessible to most elderly.

There is general agreement that memory performance declines from early to late adulthood, and that such age-related memory impairments do not involve every domain of Memory (Grady and Craik, 2000). Decrements are typically slight in implicit memory tasks, immediate memory tasks, and in many recognition memory tasks (Grady and Craik, 2000). In contrast, age-related memory losses are substantial in episodic memory tasks involving cued or free recall (Anderson and Craik, 2000; Balota et al., 2000; Grady and Craik, 2000; Nyberg et al., 2012). In this regard, it has been shown that episodic memory (Chalfonte and Johnson, 1996; Naveh-Benjamin, 2000; Naveh-Benjamin et al., 2003, 2004), and in particular the memory for face-name or face occupation associations (Naveh-Benjamin et al., 2004; James et al., 2008; Hayes et al., 2015), is markedly reduced in the elderly. However, recent elderly studies have shown that the engagement in physical activity can counteract those episodic memory losses (Zlomanczuk et al., 2006; Hayes et al., 2015). For instance, Hayes et al. (2015) showed that engagement in physical activity, is positively associated with performance on the face-name association task. However, the neuronal correlates of this effect in terms of brain activation and functional connectivity have not yet studied. Sperling et al. (2003) examined the pattern of brain activation during the encoding of face-name associations in young and elderly. The authors showed that elderly, compared to young adults, have greater activation in parietal regions but less activation in both superior and inferior prefrontal cortices and the hippocampus, a brain region known to be essential in episodic memory (Burgess et al., 2002). One may hypothesize that engagement in aerobic physical activities has a positive effect on these brain regions affecting encoding related brain activation in and functional connectivity between these brain regions. Anatomically, the hippocampus is strongly connected to prefrontal regions as medial prefrontal cortex (mPFC; Preston and Eichenbaum, 2013) which, in turn, have reciprocal connections to several thalamic nuclei that are indirectly or directly reciprocally connected to the hippocampus in monkey (Aggleton et al., 2011). Moreover, a recent fMRI study revealed functional connectivity between hippocampus, mPFC and thalamus during episodic memory retrieval in young adults (Thielen et al., 2015). Therefore, we hypothesize that face association learning (encoding) is associated with the hippocampal-thalamus-mPFC axis and that engagement in aerobic physical activity has a positive effect on activation and functional connectivity within this memory network.

There is evidence that aerobic physical activity is associated with reduced systemic inflammation (Elosua et al., 2005; Autenrieth et al., 2009). There is also evidence that age related episodic memory decline is associated with inflammation (Simen et al., 2011). An association between inflammation and memory impairment has been reported in both, rodents, and human studies (Heyser et al., 1997; Gemma et al., 2005; Barrientos et al., 2006, 2009; Hilsabeck et al., 2010; Simen et al., 2011; Harrison et al., 2014, 2015). Thus, there seems to be an interaction between physical activity, inflammation and aging related memory decline. In this regard, it has been reported that inflammation affects the functioning of the hippocampus. For instance, peripheral injection of the bacteria *Escherichia coli* – leading to increased inflammation – produces both retrograde and anterograde amnesia in 24 month old, but not 3-month-old rats for memories that depend on the hippocampus (Barrientos et al., 2006). Recent studies in human have linked hippocampal activation and functional connectivity to systemic inflammation (Harrison et al., 2014, 2015). It was shown that induced (*S. typhi* vaccination) inflammation causes a reduced medial temporal cortex glucose metabolism and selectively impaired spatial episodic, but not procedural, memory (Harrison et al., 2014). Moreover, induced inflammation blocked functional connectivity between the substantia nigra and hippocampus that occurred during novelty processing in noninflammatory states (Harrison et al., 2015). Thus, it seems that inflammation has pronounced effects on hippocampus both, in terms activation and connectivity. Therefore, we assume that inflammation is inversely related to encoding related activation and functional connectivity within the hippocampal-thalamus-mPFC axis. Interleukin-6 (IL-6) has been recognized as an active player in inflammation (Rincon, 2012). IL-6 is both an anti-inflammatory and pro-inflammatory cytokine and can be released from different cell types as for instance astrocytes, muscle or fat cells (Gruol and Nelson, 1997; Nybo et al., 2002). IL-6 released from muscle tissue during or immediately after a bout of exercise exert anti-inflammatory effects by suppressing pro-inflammation factors. For instance, elevations in skeletal muscle derived IL-6 trigger an anti-inflammatory cascade by lowering the release of pro-inflammatory cytokines (e.g., IL-1β) via the stimulation of their antagonistic receptors (Nimmo et al., 2013). Moreover, exercise-related IL-6 triggers the release of IL-10, an anti-inflammatory molecule, which directly inhibits the synthesis of different pro-inflammatory mediators, particularly of the monocytic lineage, such as TNF-*α*, IL-1α, IL-1β, IL-8, and macrophage inflammatory protein-1α (Petersen and Pedersen, 2005) At rest, the release of IL-6 from skeletal muscle is minimal, with the majority being produced from adipose tissue and leucocytes, which is thought of as pro-inflammatory (Fischer, 2006; Nimmo et al., 2013). Moreover, studies revealed that regular engagement in physical activities is associated with lower systemic IL-6 levels at rest. For instance, Elosua et al. (2005) reported a negative relation between interleukin-6 to both physical fitness and leisure time related physical activity in the elderly. Lower levels of the pro-inflammatory IL-6 may reduce the risk of adults developing neurodegenerative diseases (Laurin et al., 2001; Hamer and Chida, 2009; Middleton et al., 2010; Llamas-Velasco et al., 2015). For instance, IL-6-treated hippocampal neurons showed tau hyperphosphorylation (Quintanilla et al., 2004), a hallmark of Alzheimer’s disease. Moreover, neurons subjected to chronic IL-6 treatment exhibit increased sensitivity to NMDA receptor mediated neurotoxicity (Qiu et al., 1998). In addition, it has been shown that IL-6 can have negative effects on synaptic plasticity. For instance IL-6 affects synaptic plasticity in the CA1 region of the hippocampus by causing a marked decrease in the expression of long term potentiation (LTP), the cellular model of learning and memory (Gruol and Nelson, 1997; Tancredi et al., 2000). However, we should note that IL-6 has not only destructive but also a beneficial potential. In this regard, numerous studies provide evidence for an IL-6 involvement in neuronal survival, protection, and differentiation (Hirota et al., 1996; Gadient and Otten, 1997; März et al., 1997; Loddick et al., 1998).

In the light of the aforementioned findings, we hypothesized that aerobic physical activity does not only improve episodic memory (Hayes et al., 2015) but that this effect goes along with changed brain activation and connectivity in the hippocampal-thalamus-PFC axis which in turn is inversely related to inflammation as measured with systemic IL-6 at rest. Therefore, this cross sectional study examined the effects of aerobic physical activity engagement on the performance on a face association task and related brain activation and functional connectivity in the elderly. Moreover, we hypothesized that systemic IL-6 levels are reduced in individuals that engage in aerobic physical activity which in turn is related to the functional effects, especially those that are related to the hippocampus.

## Materials and Methods

### Subjects

Thirty-two healthy elderly, right-handed volunteers (16 males, mean age 60.4 ± 5.0 *SD*; range 52–71 years) were examined. None of the subjects reported a history of neurological or psychiatric diseases and all were free of psychotropic medication. Participants had normal or corrected-to-normal vision. Exclusion criteria were febrile illness within 7 days prior to study participation and severe somatic diseases, such as thyroid dysfunction, hypercortisolism, or adrenal dysfunction as well as diabetes mellitus type I and type II with an HBA1c > 8%, subjects with regular medication other than diabetes type 2 related medication. Written informed consent was obtained according to the local medical ethics committee.

### Procedure

Assessments were carried out during 1 day. Before scanning, each subject scored the Physical Activity Scale for the Elderly (PASE; Washburn et al., 1993) questionnaire and a blood sample was taken to define plasma levels of IL-6. Since there is strong evidence for an increased level of IL-6 immediately after a bout of exercise that last at least 90 min (Leggate et al., 2010; Nimmo et al., 2013), it is important to note that the participants had not engaged in physical exercise on the day of testing. In addition, to account for potential differences between groups, each subject performed a standard neuropsychological test battery. The neuropsychological assessment included (1) the German version of the *Auditory Verbal Learning Test* (VLMT; Lux et al., 1999) to assess verbal episodic memory, (2) the *Brief Visuospatial Memory Test-Revised* (BVMT-R; Benedict et al., 1996; Benedict, 1997) and (3) the *Paired Associates Learning* (PAL; Torgersen et al., 2011) as measures of visuospatial episodic memory. (4) the *Trail Making Test* (Tombaugh, 2004; Bowie and Harvey, 2006) version A (TMT-A, visuoperceptual abilities; Sánchez-Cubillo et al., 2009) and B (TMT-B, working memory; Sánchez-Cubillo et al., 2009) and (5) the *Intra- and Extra-dimensional Shift* (IED; Égerházi et al., 2007) to assess cognitive flexibility and executive functions as well as (6) the *Controlled Oral Word Association Test* (COWAT; Baldo et al., 2006) to assess for verbal fluency. The *Mehrfachwahl-Wortschatz-Intelligenztest* (MWT-B; Lehrl, 2005) was conducted to estimate subject’s general educational status as measurement for IQ. Both, the PASE and the neuropsychological test battery were performed before the blood sampling to ensure that the participants were not engaged in any physical activities for 90 min.

### Physical Activity Assessment

The Physical Activity Scale for the Elderly (PASE; Washburn et al., 1993) provides a measure of physical activity regarding the past 7 days and is composed of the individual engagement in activity like sports, gardening, household activity, etc. Physical activity is commonly described by the following four dimensions: (1) frequency, (2) duration, (3) intensity, and (4) type of activity (Caspersen et al., 1985). Any assessment of physical activity should ideally measure all of these dimensions and account for day-to-day variation (Warren et al., 2010). The PASE questionnaire measures all dimensions and is therefore an appropriate measurement to assess physical activity level. Since we aimed at elucidating the effects related to aerobic physical activity we used the “strenuous sport” PASE sub-score to assess the aerobic physical activity level. Based on the finding that the PASE has demonstrated good validity in a couple of evaluations as for instance peak oxygen uptake, systolic blood pressure and measurements assessing physical fitness (Washburn et al., 1993, 1999; Harada et al., 2001) we assume that in particular this subscore has the potential to measure variations in aerobic capacity.

### IL-6 Assessment

Blood samples were collected in EDTA tubes from the cubital veins between 9:00 am and 12:00 am in the fasting state and processed within 2 h by centrifugation at 1600 *g* for 15 min at RT. Plasma aliquots (500 μl) were stored in Matrix^TM^ tubes (Thermo Fisher Scientific, Inc., Waltham, MA, USA) at -80° until IL-6 determination. IL-6 was determined in duplicate with the Human IL-6 Quantikine^TM^ HS (high-sensitivity) ELISA Kit in 1:2 prediluted plasma. All readings were acquired in the dual wavelength mode at 467/650 nm on a Tecan Infinite Pro^TM^ microplate reader (Tecan, Switzerland) with the Magellan^TM^ data analysis software and corrected for background. Standard curves were generated on the basis of a four parameter logistic (4-PL) curve-fit.

### fMRI Design

**FIGURE 1:**
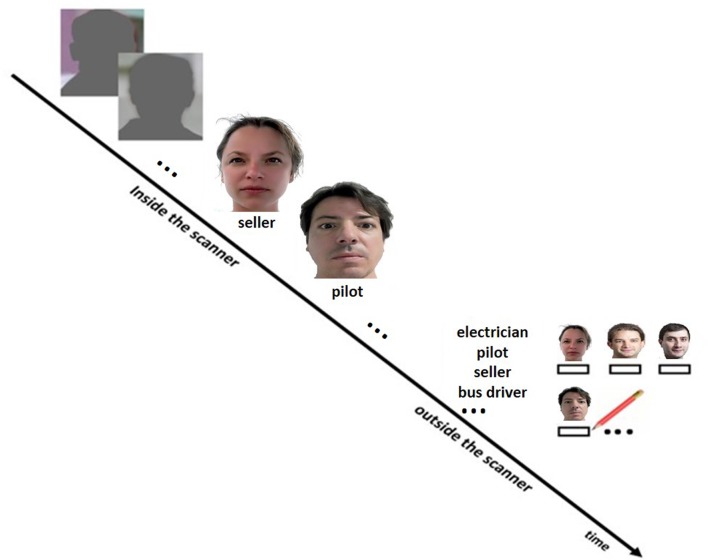
**Design of the face profession association task**. During scanning, subjects performed the face-profession association task and a visuo-motor control task. The participants were instructed to memorize the face-profession associations for a subsequent memory test and to judge whether the face fitted well with the underlined profession or not. A simple visuo-motor task was used as control condition, in which subjects were required to judge whether the ears of a shadow-masked face contour were closer to the left or the right shoulder. Thereafter, subjects performed a recall test for the associated profession outside the scanner. Therefore, the participants were provided with a list on which all professions were listed and the faces (printed on papers in A4 format) seen during scanning. The participant had to write down the associated professions below the faces.

An associative face-profession encoding task (Theysohn et al., 2013) was performed during fMRI scanning (**Figure 1**). Five blocks of an episodic memory condition (face-profession encoding task) consisting of four stimuli as described below were interleaved by five blocks of a control condition consisting of six stimuli whereby each block lasted 22.8 s. During the episodic memory condition, a series of four novel faces uniquely associated with occupational titles were shown. Each face with its associated occupational title underneath was displayed at the center of the screen for 5.7 s. Subjects were instructed to memorize face-profession associations for a subsequent memory test and to judge whether the face fitted well with the underlined profession or not. A simple visuo-motor task was used as control condition, in which each block (as in the episodic memory condition) started with the presentation of a brief instruction for 2.0 s, and followed by showing a series of six shadow-masked face contours with the presentation time of 3.8 s each. Subjects were required to judge whether the ears of a shadow-masked face contour were closer to the left or the right shoulder. We have chosen an “active” control task to avoid mental processes that are related to memory formation. In other words, during the visuo-motor task the participants could not spend effort to remember the faces and related occupations in order to improve memory. After the fMRI task subjects performed a recall test for the associated profession outside the scanner whereby they were presented with all the faces (printed on papers in A4 format) and had to write down the associated profession. To simplify this task, participants were also provided with a list on which all professions were listed. Stimuli, consisted of 20 portraits (half males) and 20 familiar professional names, were standardized according to several criteria, such as no strong emotional facial expression, direct gaze contact, no glasses, no beard, no headdress, etc. The order of stimuli presentation was randomized for each subject. Length of familiar professional names ranged from 7 to 15 letters (mean length ±*SD* = 10.65 ± 2.77). For the control condition, 30 color photographs showing shadow-masked face contours (with shoulders) served as stimuli.

### MRI Data Acquisition

Image acquisition was performed on a whole-body 7 T MR system (Magnetom 7T, Siemens Healthcare, Germany) using a 32-channel Rx/Tx head coil (Nova Medical, Wilmington, MA, USA). We used a T1 weighted MPRAGE sequence for structural image acquisition with a TR = 2.5 s, TE = 1.44 ms, flip angle = 6°, slice thickness = 0.7 mm, resolution = 0.7 mm^3^, FOV 236 mm × 270 mm. Functional volumes were acquired using a T2^∗^-weighted gradient echo 3D EPI sequence with ascending slice acquisition order, acceleration factor 8 (GRAPPA *R* = 4^∗^2), TR = 3.0 s, TE = 20 ms, flip angle = 15°, matrix size 192^∗^192^∗^96, slice thickness = 1.5 mm, resolution = 1.5 mm^3^, and a FOV of 288 mm × 288 mm.

### fMRI Data Analysis

The native structural T1 images were segmented into gray and white matter components. The output of the segmentation was then used to create a group specific template in SPM8 by using diffeomorphic anatomical registration through exponentiated lie algebra (DARTEL), which is registered to the Montreal Neurological Institute (MNI) space. Functional images were realigned, and the individual mean images were coregistered with the corresponding structural MRI by using normalized mutual information optimization. Then, the functional images were spatially normalized and transformed into a common space (group specific DARTEL template), as well as spatially filtered by convolving the functional images with an isotropic 3D Gaussian kernel of 6 mm FWHM. Regressors of interest were formed by creating a box-car function for both conditions (face-profession task/control task) convolved with the canonical hemodynamic response function. On the first level, a GLM was conducted with these two regressors, together with the six motion parameters derived from realignment procedure.

To investigate the effects of aerobic physical activity on the memory network, we performed psychophysiological interaction (PPI) analyses embedded in SPM8 (Friston et al., 2007). First eigenvariate values were extracted (physiological factor) from 5-mm spheres centered around the maxima within the clusters indicative of significant main effects (face-profession task > control task). A box-car function (weighted with +1 for face-profession and -1 for the control condition) was temporally convolved with the canonical hemodynamic response function (psychological factor). An interaction factor (PPI) was calculated as an interaction term of physiological and psychological factors. For each seed region, a first level GLM was conducted including the face-profession and control task regressors, the PPI regressors (physiological, psychological, and interaction factors) as well as the six motion regressors derived from realignment procedure during preprocessing of the functional scans.

### Activation Analysis (Main Effect of Memory)

To assess the main effect of memory encoding, the subject-specific contrast images (face-profession condition over control condition) from all participants (*N* = 32) were used as inputs for the second-level random effects analysis. The results of the second level random effects analyses were thresholded at *P* = 0.001 and thereafter cluster-size statistics were used as test statistic. Only clusters at *P* ≤ 0.05 (family-wise error corrected for multiple comparisons) were considered significant.

### Activation Analysis (Effects of Aerobic Physical Activity)

The subject-specific contrast images (face-profession condition over control condition) were used as inputs for the second-level random effects analysis. Age, gender, and MWT-B IQ scores were included in the model as covariates of no interest. We did a GLM analyses in SPM8 to probe differences in brain activation due to aerobic physical activity [aerobic (+) group vs. aerobic (-) group]. Given the prior findings regarding the hippocampus, a bilateral hippocampus region of interest (ROI) was defined by means of the WfU-Pickatlas (Maldjian et al., 2003, 2004) as reduced search space. Thus we performed, whole brain and ROI (hippocampus) analyses. The outcomes of the second level group analyses were thresholded at *P* = 0.001 and thereafter cluster-size statistics were used as test statistic. Only clusters at *P* ≤ 0.05 (family-wise error corrected for multiple comparisons) were considered significant.

### Functional Connectivity Analysis

We conducted separate PPI analyses (mPFC and precuneus), based on the outcome of the initial activation analyses (main effects). The subject-specific contrast images for the interaction term (PPI.ppi) were used as inputs for the second-level group analyses [aerobic (+) vs. aerobic (-)] with age, gender, and MWT-B IQ scores as covariates of no interest. As previous, we performed a whole bran and ROI analysis (hippocampus). The results of the second level group analyses were thresholded at *P* = 0.001 and thereafter cluster-size statistics were used as test statistic. Only clusters at *P* ≤ 0.05 (family-wise error corrected for multiple comparisons) were considered significant.

### Correlation Analysis

**FIGURE 2:**
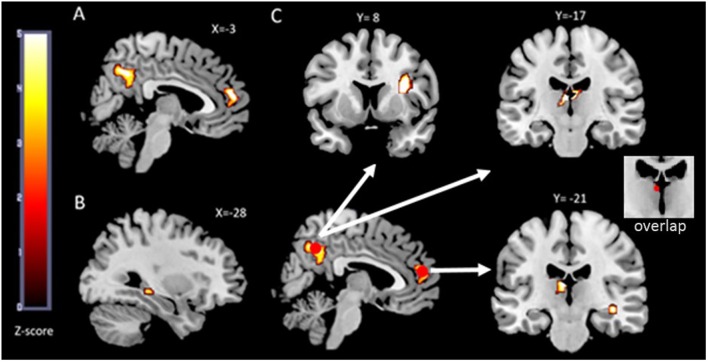
**fMRI results**. The functional maps are overlaid on the MRIcron template brain (ch256). In **(A)** an illustration of the two brain regions that showed a main effect (face-profession task over control task) in the *whole group* analysis is depicted. Both, the precuneus cluster and the mPFC cluster extend into both hemispheres and served as seed regions in the functional connectivity analyses. In **(B)** the left hippocampus region that was more activated in the aerobic (+) group compared to the aerobic (-) group is illustrated. The outcome of the performed psychophysiological interaction (PPI) analyses is depicted in **(C)**. The two red circles are the seed regions which are spheres of 5 mm centered around the maxima within the clusters depicted in **(A)**. The white arrows indicate increased functional connectivity’s between precuneus and thalamus, precuneus and right insula, mPFC and right hippocampus as well as between mPFC and left thalamus in the aerobic (+) group.

Pearson’s partial correlations analyses were performed by means of SPSS (IBM 21) software, with age, gender and MWT-B IQ score as variables of no interest. To assess the relations between brain activation/connectivity parameters and IL-6, we extracted the mean beta values of the clusters that revealed a significant effect of aerobic physical activity. For this purpose, we used MarsBaR toolbox to create separate masks of the significant clusters (see **Figures 2B,C**) which in turn were used in the REX toolbox to extract the analyses specific (PPI mPFC, PPI precuneus, or activation) mean beta-values of each cluster for all subjects. The mean *b*-values of the fMRI (activation/PPI) clusters and IL-6 concentration were then used for Pearson’s partial correlation analyses with Benjamini and Hochberg (1995) correction for multiple testing.

## Results

### Characteristics of the Sample

We grouped the subjects according to the PASE subscore “strenuous sport.” Subjects in the aerobic (+) group scored on average 9.01 (*SD* = 4.7) whereas the subjects in the aerobic (-) group did not engage in any strenuous physical activities. No significant differences were found on relevant group characteristics, such as age and gender. However, the aerobic (-) group revealed a higher MWT-B IQ score compared to the aerobic (+) group (**Table 1**). Regarding the neuropsychological assessment, we found that the aerobic (+) group performed better on the TMT-A/B and the COWAT (**Table 2**). With respect to the fMRI memory task, we found a significant difference in performance (number of correct remembered face-profession associations) between the aerobic (+/-) groups. The aerobic (+) group revealed a significantly increased memory performance compared to the aerobic (-) group (see **Table 1**). This effect remained stable if corrected for MWT-B score, age, and gender.

**Table 1:** Group differences in the aerobic (+/-) groups are depicted.

	Aerobic physical activity		*P*
	Aerobic (-)	Aerobic (+)	
	Mean (*SD*)	Mean (*SD*)	
**Demographics**			
Gender	9 M/7 W	7 M/9 W	0.480
Age	61.19 (4.87)	59.75 (5.19)	0.426
MWT-B (IQ score)	116 (13.52)	106.69 (11.53)	0.045^∗^
**Blood parameter**			
IL-6 (pg/ml)	1.98 (1.45)	0.89 (0.59)	0.009^∗^
**fMRI-Task**			
Face-professional task (remembered items)	2.62 (1.82)	5.62 (3.57)	0.006^∗^

*In the top relevant group characteristics (age, gender, and IQ) are shown. Below the group characteristics, the group differences of the parameters of interest (blood parameter and fMRI task) are depicted. ^∗^Significant*.

**Table 2:** Group differences in the aerobic (+/-) groups are depicted.

	Aerobic physical activity		*P*
	Aerobic (-)	Aerobic (+)	
	Mean (*SD*)	Mean (*SD*)	
**Neuropsychology**			
VLMT-recognition (remembered items)	10.50 (2.65)	10.19 (4.26)	0.805
VLMT-delayed recall (remembered items)	8.53 (2.53)	9.12 (3.13)	0.570
BVMT-recognition (remembered items)	9.81 (1.97)	10.63 (1.70)	0.223
BVMT-delayed recall (remembered items)	7.5 (3.03)	9 (2.53)	0.139
PAL-stages completed	7.73 (0.5)	8 (0.0)	0.104
TMT-A (time)	40.19 (10.14)	31.88 (6.81)	0.011^∗^
TMT-B (time)	99.44 (26.41)	80.56 (22.69)	0.038^∗^
IED-stages completed	14.20 (5.58)	18 (16.99)	0.408
COWAT-correct produced	43.56 (11.33)	53.94 (12.53)	0.020^∗^

*The aerobic (+) group scored better on tests assessing visual processing speed (TMT-A), working memory (TMT-B) and verbal fluency (COWAT) but not on tests assessing verbal LTM (VLTM), visuospatial LTM (BVMT-R/PAL) or cognitive flexibility/executive functions (IED). ^∗^Significant*.

Subjecting the blood parameter scores (IL-6) to a ANOVA a statistical significant effect of aerobic physical activity on IL-6 was observed (*F*(1,30) = 7.70, *P* < 0.009) when dividing groups according to their aerobic physical activity level (+/-). This effect remained stable if corrected for MWT-B score, age and gender. A *post hoc t*-test revealed that the aerobic (+) subjects had significantly lower levels of IL-6 compared to the aerobic (-) group (**Table 1**).

### MRI Analyses

#### fMRI (Activation)

Whole brain analysis revealed a *main effect* of memory encoding in the bilateral precuneus (maxima at MNI = -2 -61 40 FWE corrected, *P* < 0.03) and bilateral medial prefrontal cortex (maxima at MNI = -6 48 6 FWE corrected, *P* < 0.05; **Figure 2A**).

While the whole-brain analysis did not reveal significant differences between the aerobic + vs - group, the ROI analysis, however, revealed significantly increased activation in the left hippocampus (maxima at MNI = -29 -33 -6, FWE corrected, *P* < 0.03) in the aerobic (+) group (**Figure 2B**).

#### Functional Connectivity

Seed ROI mPFC (**Figure 2C**): The whole brain analyses revealed a stronger mPFC-left thalamus functional connectivity (maxima at MNI = -3 -17 13, FWE corrected, *P* < 0.02) in the aerobic (+) group compared to the aerobic (-) group. Regarding the ROI approach, an increased mPFC-right hippocampus functional connectivity (maxima at MNI = 37 -21 -11, FWE corrected, *P* < 0.05) was found in the aerobic (+) relative to the aerobic (-) group.

Seed ROI precuneus (**Figure 2C**): In the aerobic (+) group a pattern of increased functional connectivity was found comprising the bilateral thalamus (maxima at MNI = -4 -16 10 FWE corrected, *P* < 0.01) and the right insula (maxima at MNI = 32 8 17 FWE corrected, *P* < 0.02) when compared to the aerobic (-) group.

### Correlation Analyses

The functional parameter that revealed a significant association with IL-6 (see **Table 3**) were the functional connectivity between mPFC/hippocampus and precuneus/insula. We found that mPFC-hippocampal functional connectivity correlated negatively with IL-6 (*r* = -0.46; *P* = 0.009). As did the precuneus-insula functional connectivity (*r* = -0.45; *P* = 0.011). In line with lesser inflammation in the presence of better connectivity within the memory network, we also found a negative correlation between hippocampal activation and IL-6 concentrations (*r* = -0.39; *P* = 0.033), which, however, did not survive the Benjamini and Hochberg correction for multiple comparisons.

**Table 3:** Correlation matrix of aerobic physical activity effects on fMRI parameter (activation and functional connectivity) and IL-6 concentration.

	IL-6
**fMRI (activation)**	
Left hippocampus	*r* = -0.39^∗^
**fMRI (connectivity)**	
mPFC to thalamus	*r* = -0.27
mPFC to hippocampus	*r* = -0.46^∗∗^
Precuneus to thalamus	*r* = -0.33
Precuneus to insular	*r* = -0.45^∗∗^

*The black correlations indicate the Benjamini and Hochberg corrected correlations*.

*^∗^Uncorrected significant*.

*^∗∗^Benjamini and Hochberg corrected significant*.

## Discussion

The present study aimed to combine immunological and functional imaging parameters to investigate multifactorial protective mechanisms of physical activity in healthy elderly. More precisely, we assessed the potential impact of the engagement in aerobic physical activity on changes in a network of brain regions mediating episodic memory functions and the associations to inflammation. As a marker for inflammation we indexed IL-6 since this cytokine displays the most marked response to acute exercise compared to other inflammation marker as for instance TNF-R, TNF alpha, IL 1 beta, IL-1ra, or IL-10 (Petersen and Pedersen, 2005).

Behaviorally, the aerobic (+) group had an elevated memory performance for face-profession associations compared to the aerobic (-) group, which is in line with the recent finding of Hayes et al. (2015). This finding was paralleled by better scores on tests assessing visual processing speed (TMT-A), working memory (TMT-B), and verbal fluency (COWAT) but not on tests assessing verbal episodic memory (VLTM), visuospatial episodic memory (BVMT-R/PAL) or cognitive flexibility/executive functions (IED). Note, we found only in the face-association task but not on the other episodic memory tasks an effect of physical exercise. This results are only partly in line with the findings of Hayes et al. (2015), which showed also no effect on verbal episodic memory but on visuospatial episodic memory. This may be related to methodological differences since Hayes et al. (2015) measured the level of physical activity via accelerometry. Moreover, Hayes et al. (2015) showed that the face-association task seems to be more sensitive to physical activity in older adults, since physical activity level accounted for 29.6% of the variance in the face-association task compared to 13.3% of the variance on the neuropsychological tests of visuospatial episodic memory. Regarding brain activation (main effect), we found face-profession encoding related activation in the medial precuneus and the mPFC. While in line with other studies, we also found an activation in ventral temporal areas and lateral parietal as well as lateral frontal areas, these clusters failed to reach significance in the whole brain analysis, mirroring the findings from Theysohn et al. (2013), which used the same experimental paradigm and 7 Tesla scanning in healthy young adults. It is important to note, that dissimilar to Theysohn et al. (2013), there was signal dropout in the ventral temporal cortices, reducing the size of the activation cluster in the hippocampus so that the hippocampal clusters did not reach significance in the whole brain analyses. As aforementioned, based on anatomical connections and previous fMRI reports we assumed the mPFC-thalamus- hippocampus axis to be involved in the given memory task and that the engagement in aerobic activity increases functional connectivity in this axis. Therefore, we used the mPFC region as seed region in the PPI analyses. The precuneus is assumed to be involved in visual imagery and working memory occurring in episodic memory (Fletcher et al., 1996; Halsband et al., 1998; Cavanna and Trimble, 2006). Given the rich anatomical connectivity to several thalamic nuclei (Cavanna and Trimble, 2006) and reports of aging related changes in precuneus functions (Sperling et al., 2003; Gould et al., 2006; Mevel et al., 2011; Yang et al., 2014; Kleerekooper et al., 2016), we used the precuneus region as a second seed region in the PPI analyses. Note, since our preliminary hypothesis was focused on the mPFC-thalamus-hippocampus axis, the analyses regarding the precuneus cluster have an exploratory characteristic. In the following paragraphs, the effects of self-reported engagement in aerobic physical activity on brain activation/functional connectivity and the relation to inflammation will be discussed.

Whereas behavioral effects of physical activity/fitness on episodic memory have been frequently studied its relation to functional parameter as memory related brain activation or functional connectivity are still poorly understood. Here, we provide first evidence that engagement in aerobic physical activity is associated with episodic memory related brain activation and functional connectivity. More precisely, we found that the aerobic (+) group revealed stronger BOLD *activation* in the left hippocampus and a stronger *functional connectivity* between mPFC and left thalamus/right hippocampus during memory encoding. The thalamus has been described as an important structure regarding mPFC-hippocampus “communication” during memory processes. Evidence from human imaging studies as well as animal data revealed that the mPFC-thalamus-hippocampus axis is strongly associated with memory encoding (Xu and Südhof, 2013) memory consolidation (Thielen et al., 2015) and memory retrieval (Aggleton and Brown, 1999; Davoodi et al., 2009, 2011; Aggleton et al., 2010; Loureiro et al., 2012). Here, we show for the first time, that engagement in aerobic physical activity is associated with increased activation and functional connectivity in the mPFC-thalamus-hippocampal axis when elderly learn new face-occupation associations. Unfortunately, we could not observe a relation between the functional effects and performance on the face-profession task. However, in contrast to event related designs, blocked designs (as used here) are not able to distinguish between successful vs unsuccessful encoding processes.

In addition, we found that the aerobic (+) group revealed a stronger *functional connectivity* between precuneus and bilateral thalamus/left insula. The precuneus is assumed to be involved in visual imagery occurring in episodic memory (Fletcher et al., 1996; Halsband et al., 1998; Cavanna and Trimble, 2006). Interestingly, both seed regions (precuneus and mPFC) revealed increased functional connectivity to the thalamus that overlaid in the midline/dorsomedial thalamus. Therefore, the precuneus/thalamus connectivity might reflect support of the mPFC-thalamus-hippocampus axis with information regarding the visual representation of the memory. The insula, has been functionally divided into a posterior, ventroanterior and dorsoanterior part (Wager and Feldman-Barrett, 2004; Chang et al., 2013). In the present study, we found precuneus functional connectivity to the dorsoanterior insula, a region that is commonly activated in tasks that require executive control of attention, including those that require manipulation of information in working memory (Wager and Smith, 2003), shifting attention and response inhibition (Wager et al., 2004). Hence, the underlying function of the precuneus/insula connectivity may be related to executive manipulations of information in the working memory. To summarize, as hypothesized, the engagement in aerobic physical activity increased activation and functional connectivity within the mPFC-thalamus-hippocampus axis in the elderly. Interestingly, we found that the engagement in aerobic physical activity increases also task related functional connectivity in a precuneus-insula network that appears to interact with the mPFC-thalamus-hippocampus axis via the thalamus. Thus, we provide initial evidence that the thalamus has the potential to connect different networks, probably involved in different aspects of episodic memory encoding, a function that is boosted due to the engagement in aerobic activity.

Finally, we assessed whether systemic IL-6 concentrations are related to the functioning within the episodic memory network. In this regard, Harrison et al. (2014, 2015) have shown that induced inflammation causes a reduction in hippocampal glucose metabolism and functional connectivity during memory related processes. In the present study, we found an inverse relation between mPFC-hippocampus functional connectivity and circulating IL-6. In addition, a negative correlation between hippocampal activation and IL-6 could be observed. However, this correlation did not survive the Benjamini and Hochberg correction for multiple comparisons. Regarding the functional connectivity between mPFC and thalamus there was no relation to IL-6 which may be related to the heterogeneity of IL-6 distribution within the brain. For instance, rodent studies revealed high levels of IL-6 mRNA and IL6 receptor mRNA expressions in some brain regions, including the hippocampus, if compared to other brain regions (Schobitz et al., 1993; Gadient and Otten, 1994; Aniszewska et al., 2015). Together, we found that the functioning in the mPFC-thalamus-hippocampus axis is negatively related to inflammation, extending the previous findings of Harrison and colleagues to episodic memory processes in the elderly. Interestingly, we found also an inverse relation between IL-6 and the functional connectivity between precuneus and insula. This is in line with recent studies that showed an association between inflammation and the insula in human (Hannestad et al., 2012; Labrenz et al., 2016). For instance, Labrenz et al. (2016) induced inflammation with lipopolysaccharide in healthy young adults and measured inflammation produced changes in resting state functional connectivity. They found a strong reduction between insula-precuneus functional connectivity, which was most pronounced for the anterior part of the insula. In the present study, we reveal for the first time that encoding related functional connectivity to the insula is associated to inflammation as measured with IL-6. Altogether, we provide new evidence that self-reported engagement in aerobic physical activity predict the strength of brain activation and functional connectivity in an episodic memory network composed of hippocampus, mPFC, thalamus, precuneus, and insula. Within this network, it appears that the circulating inflammatory marker IL-6 is inversely related to mPFC/hippocampal and precuneus/insula functional connectivity extending previous research that showed high affinity of inflammation on these brain regions. With respect to memory performance, we could not observe a relation between the amount of remembered associations and IL-6 levels, which may be attributed to the small sample size.

Some limitations should be acknowledged in this study. First, due to susceptibility artifacts, there was signal dropout in the ventral temporal cortices which reduced the size of the activation clusters (main effect) in this areas. Thus we cannot rule that these clusters would have reached significance in the absence of this artifact. Second, we did not assess the individual levels of physical fitness. Therefore, the findings we report here in relation to aerobic physical activity cannot transferred directly to aerobic capacity (fitness). For instance, a participant that reported 3 h of jogging may have run a distance that was much less than that of another participant that reported also 3 h of jogging. Moreover, physical activity is socially desirable behavior that might be overreported because of a social desirability bias. Third, this study had a non-randomized design so any number of third variables could have influenced the results. Fourth, our preliminary hypothesis did not include the involvement of the precuneus in the given task rendering the second PPI analysis more exploratory. Furthermore, we used IL-6 only as a measure of inflammation which does not represent the whole complexity going on in inflammatory processes. To date, many different markers of inflammation have been discovered. Therefore, to get a more comprehensive understanding, future studies should also indexing other inflammatory marker as for instance NF Kappa B, TNF alpha, and IL-10. In addition, we cannot determine whether the IL-6 effect is related to cells in the CNS (e.g., astrocytes) or cells in the periphery as for instance muscle or fat cells. In this regard, Nybo et al. (2002) showed that IL-6 release from the CNS is increased after a bout of exercise. However, the net release of IL-6 from the CNS appears to be manyfold lower than that released from muscles (Steensberg et al., 2001; Nybo et al., 2002). Future studies should use controlled experimental designs (e.g., Nybo et al., 2002) to determine the effects of physical activity on CNS released IL-6.

## Conclusion

We assessed the impact of the engagement in aerobic physical activity on immunological and functional imaging parameters in healthy elderly in a between-subject cross-sectional design. We replicated prior findings regarding better memory functioning and decreased IL-6 concentration in aerobic active elderly subjects. In addition, we provide new evidence for an effect of aerobic physical activity on episodic memory related activation and functional connectivity. Moreover, we demonstrate that episodic memory related hippocampal and insula functional connectivity is inversely related to circulating IL-6 extending previous findings of inflammation effects on network properties. Future studies should try to replicate the current findings in a prospective intervention set-up to assess the impact of physical activity on the given parameters and their relations over time.

## Author Contributions

J-WT (data acquisition, analysis, and writing), CK (data acquisition and writing), BM (writing), IR (Neuropsychology), JG (Interleukin 6), BB (writing), SM (fMRT sequences), DN (MRT, supervisor), JW (supervisor), and IT (analysis, writing, and supervisor).

## Conflict of Interest Statement

The authors declare that the research was conducted in the absence of any commercial or financial relationships that could be construed as a potential conflict of interest.
